# Tillage Effects on Selected Soil Physical Properties in a Maize-Bean Intercropping System in Mwala District, Kenya

**DOI:** 10.1155/2014/497205

**Published:** 2014-11-03

**Authors:** Anne Karuma, Peter Mtakwa, Nyambilila Amuri, Charles K. Gachene, Patrick Gicheru

**Affiliations:** ^1^Department of Soil Science, Sokoine University of Agriculture, P.O. Box 3151, Morogoro, Tanzania; ^2^Department of Land Resource Management and Agricultural Technology (LARMAT), University of Nairobi, P.O. Box 29053, Nairobi, Kenya; ^3^National Agricultural Research Laboratories (KARI), P. O. Box 14733, Nairobi, Kenya

## Abstract

A field study was carried out to evaluate the effects of tillage practices on soil physical properties in Mwala district, Eastern Kenya, during the long rains (LR) and short rains (SR) of 2012/13. The treatments were disc ploughing (DP), disc ploughing and harrowing (DPH), ox-ploughing (OX), subsoiling-ripping (SSR), hand hoeing with tied ridges (HTR), hand hoeing only (H). These were investigated under three cropping systems of sole maize, sole bean, and maize-bean intercrop in a split-plot design with four replications. Soil physical properties were monitored at different weeks after planting (WAP) throughout the growing seasons. A four-season average shows that soil moisture content was significantly (*P* < 0.05) higher in OX > SSR > DPH > H > HTR > DP with values ranging from 13.1 to 14.1%. Soil surface roughness and crust strength varied significantly (*P* < 0.05) over time within the growing seasons, between the tillage practices, and across the different seasons with values ranging from 26 to 66% and 1.21 to 1.31 MPa, respectively. Tillage practices and cropping systems did not significantly affect bulk density, porosity, or *K*_sat_ values. It is apparent that long term tillage experiment (>4 seasons) would be required to detect changes in soil physical properties as a result of the soil management practices.

## 1. Introduction

Smallholder agriculture in semiarid areas is hampered by accelerated soil erosion, induced soil moisture deficits, soil fertility depletion, and soil crusting and compaction [1]. The soil crusting and compaction problem is attributed to inherent soil properties and poor tillage practices [2]. The semiarid areas of Kenya are characterized by temporal and spatial variability of rainfall which is not suitable for sustainable rainfed agriculture [1, 3]. Thus, resource-poor farmers in these areas who entirely rely on rainfed agriculture are subject to various hydrological constraints. The rainfall occurrence is bimodal with two distinct rainy seasons: the short and long rains. In Kenya's semiarid areas, the short rains (October to December) are more reliable and are evenly distributed and more often support plant growth [4]. However, very high soil moisture deficits experienced in these areas usually result in significant decreases in crop yields under rainfed conditions. Deficit of soil moisture in these areas is attributed to low infiltration rates caused by surface sealing, crusting, low organic matter content, and subsequent high runoff rates [1, 5, 6]. Soil moisture conservation under such conditions requires appropriate tillage practices that not only improve rainwater infiltration but also conserve adequate soil moisture for plant growth [7].

Soil tillage, as a necessary practice in crop production, can affect the soil physical properties that are important for plant growth [8, 9]. Improvements of root penetration, water infiltration and soil moisture storage, weed control, and supply of nutrients from rapid decomposition of organic matter are considered the most beneficial contributions of tillage to crop production [10–12].

Conventional tillage involves the mechanical soil manipulation of an entire field, by ploughing (inverting the soil) followed by one or more harrowings. The degree of soil disturbance depends on the type of implement used, the number of passes, type of soil, and intended crop type [13]. The most common conventional tillage practiced in Kenya involves the use of hand hoes, ox-drawn mould board ploughs, tractor-drawn disc ploughs, and harrows combined with straw collection and burning during land preparation [2, 14]. During the operation, the soils are cut, inverted, and pulverized burying most of the residues underneath [14]. Consequently, in many areas, especially semiarid areas, conventional agriculture has led to a decline in crop yields and profitability when compared to those realized from areas with higher rainfall [15].

Other sustainable techniques advocated in the semiarid areas for soil and water conservation have included tied ridging, cover cropping, mulching, subsoiling and ripping [16, 17]. These techniques are referred to as conservation tillage and have the potential of soil moisture retention and mitigation of intraseasonal dry spells that often result in low productivity and crop failure, reduce soil evaporation, and enable organic matter build-up which enhances water holding capacity of the soil [18–20].

Conservation tillage practices are particularly important for increasing the productivity of water through reducing runoff and evaporation and improving soil water storage [18]. In Kenya, promotion of animal-drawn conservation tillage tools such as rippers and subsoilers among smallholder farmers has resulted in improved water productivity and crop yields [21, 22]. Although conservation tillage is highly advocated, there is strong evidence that this kind of tillage may not be good with soils prone to surface crusting and sealing, a characteristic of most of the soils in the semiarid areas of Kenya [23–26]. Therefore, the local biophysical conditions in the smallholder farming systems in these semiarid areas need to be considered and deliberate adaptation efforts made.

The success of any tillage practices is directly related to the improvement of the soil physical properties which in turn may affect the growth and yield of crops due to the different soil conditions created. In a review on the effect of soil physical properties on soil moisture, Strudley et al. [27] showed that bulk density, porosity, soil surface sealing and crusting, surface roughness, hydraulic conductivity, and infiltration rates were highly influenced by different tillage. Therefore, the choice of any tillage system is too critical for maintenance of the soil physical properties necessary for crop growth [28]. However, the resulting effect of tillage on selected physical properties depends on the site-specific biophysical environment such as seasonal variability in rainfall, inherent soil fertility status, or the prevailing climatic conditions [29]. Few studies have measured these soil physical properties, especially in semiarid Eastern Kenya, hence the essence for the study reported herein.

Recent efforts have focused on minimum tillage practices as a soil and water conservation measure [30, 31]. Nonetheless, maize-legume cropping systems are popular in improving land use efficiency and economic returns. In order to exploit these practices, there is a need to understand the influence of tillage and cropping systems on selected soil physical properties in dryland cropping systems for sustained productivity. The objective of this study was to investigate the effect of selected tillage practices and cropping systems on selected soil physical properties in semiarid Mwala district, Eastern Kenya. Specifically, their effects on soil moisture, soil surface roughness, crust strength, saturated hydraulic conductivity, bulk density and porosity.

## 2. Materials and Methods

### 2.1. Study Site Description

The study was conducted in Mbiuni location, Mwala district, Machakos County, Eastern Kenya (1°15′S, 37°25′E). The area is characterized by low, erratic, and poorly distributed bimodal rainfall that makes crop production difficult under rainfed conditions. The long rains commence in mid-March and end in May while short rains start in mid-October and end in late November. The mean annual rainfall for Mwala district is 596.7 mm [32]. Maize and beans dominate household consumption. Pulses are grown in the district and the predominant ones are beans, pigeon peas, cowpeas, green grams, and chickpeas. Soil chemical and physical properties at the site are given in Table 1. The study site was previously under beans and the slope was <2%.

### 2.2. Experimental Design and Layout

The trials were established in 2012 and ran for four seasons during the long (LR) (March–May) and short rains (SR) (October–December) (i.e., LR 2012, SR 2012, LR 2013, and SR 2013). The treatments consisted of six tillage practices: disc ploughing (DP), disc ploughing and harrowing (DPH), ox-ploughing (OX), hand hoeing with tied ridges (HTR), hand hoeing only (H) and subsoiling-ripping (SSR). The cropping systems treatments were sole maize, sole bean, and maize-bean intercrop. The treatment factors were arranged in a split-plot design with tillage practices as the main plots and the cropping system as the subplots and replicated four times in plot sizes of 25 m^2^. Time in weeks after planting (WAP) and season were considered as experimental factors to test the changes within a growing season and across the different cropping seasons.

### 2.3. Soil Properties Measurements

Soil moisture was monitored from crop emergence to harvesting at depths of 0–20 cm and 20–40 cm using the gravimetric method [33]. Monitoring of soil moisture was done at the 0–40 cm depth due to the concentration of active roots at this level and less below this depth range. Soil surface roughness was measured immediately after the tillage operations and before weeding was done. Readings were taken from three randomly selected positions in each tillage plot. A microrelief meter similar to that described by Kuipers [34] was used to measure surface roughness.

Crust strength (penetration resistance) was measured at the soil surface (0–10 cm depth) using a hand-held penetrometer (Eijkelkamp equipment type 1B) in three seasons (SR 2012, LR 2013, and SR 2013). Ten soil crust strength measurements were taken at randomly selected positions in each plot. The penetrometer springs and cone sizes were adjusted depending on the soil strength. Crust strength was calculated as

(1) $$\begin{array}{c} \text{CR}=I\;\times \;\left(\frac{\text{Cs}}{\mathrm{AC}}\right), \end{array}$$

where CR is the cone resistance (N cm^−2^), *I* is the impression on the scale (cm), Cs is the spring constant (N cm^−1^), and AC is the area of the cone (cm^2^).

Saturated hydraulic conductivity (*K*_sat⁡_) determinations were done in the laboratory using the constant head method as described by Klute and Dirksen [35]. Undisturbed soil core rings (5 cm depth, volume 98.13 cm^3^) collected from the field were carefully trimmed to the size of the core ring. A piece of muslin cloth held with a rubber band was used on one side of the core ring to protect the soil from spilling but to allow water to pass through. The samples were then saturated for 24 hours before water percolation tests were done. The volume of water that passed through the soil sample was measured and recorded until a constant average was achieved. The *K*_sat⁡_ was then calculated as

(2) $$\begin{array}{c} K_{\mathrm{sat}}=Q\times \frac{L}{\left(A\times T\times H\right)}, \end{array}$$

where *Q* is the discharge or percolate through the soil (cm^3^ or mL), *L* is the length of the soil core (cm), *A* is the cross-sectional area of the soil core (cm^3^), *T* is the time taken (hours), and *H* is the hydraulic head difference (cm).

The bulk density was determined using undisturbed core samples from each plot. Soil core samples were carefully trimmed at both sides to the size of the core ring. The soil core was then oven-dried at 105°C to a constant mass and then weighed. Bulk density was then calculated as the mass of the dry soil divided by the core ring volume. Total porosity was then calculated from the bulk density as

(3) $$\begin{array}{c} \text{Total}\;\text{porosity}=\left(1-\frac{\rho_{b}}{\rho_{s}}\right), \end{array}$$

where *ρ*_*b*_ is the bulk density and *ρ*_*s*_ is the average particle density (2.65 Mg/m^3^). The *K*_sat⁡_, bulk density, and total porosity were determined at one or two intervals in a growing season (the beginning of the season (3 WAP) and at harvest).

### 2.4. Crop Management

A dryland maize variety (DH 02) and beans (rose coco: GLP 2) were used as the test crops. These crops were planted in rows in 25 m^2^ plots. The maize was planted at a spacing of 90 × 30 cm in pure stands while the bean plants were planted at a spacing of 45 × 15 cm. In the tied ridging plots, maize and beans were planted in the same row but in alternating hills. Thinning was done to a single plant per hill for maize and two plants for the legume, four weeks after germination.

In each cropping system, the nitrogen was applied at 60 kg N ha^−1^ (DAP 18 : 46 : 0) at planting to the maize and additional 60 kg N ha^−1^ (CAN 26 : 0 : 0) was top-dressed when maize was knee-high (4 WAP). The beans were inoculated with Bio-Fix⁡^©^ biofertilizer before sowing. All plots were hand-weeded using a hand hoe as practiced by the farmers during the cropping periods. No crop rotation was done during the study.

### 2.5. Statistical Analysis

In analysis of the soil properties, analysis of variance (ANOVA) with repeated measures was used using Genstat 14th Edition statistical software. Mean separation was done using LSD at 5% level of probability where the ANOVA* F*-values were significant.

## 3. Results and Discussion

### 3.1. Soil Moisture Content as Affected by Tillage Practices and Cropping Systems between Season and Depth

Tillage practices resulted in significant differences in moisture content in LR 2012 (*P* = 0.019), LR 2013 (*P* = 0.003), and SR 2013 (*P* = 0.001) but not in SR 2012 (*P* = 0.158) (Table 2). However, in SR 2012, higher moisture levels were observed in OX (15.3%) and SSR (14.4%). The OX and SSR plots maintained high moisture levels in the SR 2012 and LR 2013. Subsoiling-ripping (SSR) and ox-ploughing (OX) enhance water penetration into deeper soil layers and allow deeper rooting of crops that benefit from stored water and nutrients as they exploit larger soil volumes. Steiner and Rockstrom [36] made similar observations. In LR 2012, the HTR plots had high moisture levels (15.6%) due to the microbasin formation that traps rainwater and allowed more time for infiltration and storage. In contrast to LR 2012, HTR had the lowest moisture (8.42%) in the LR 2013 season.

Similar findings are supported by Guzha [37], who found that the higher moisture was stored in ridges and this was associated with higher roughness resulting from ridge configuration. Tied ridges create rectangular basins between ridges, which increase surface retention capacity and decrease runoff, thus improving soil moisture content and ultimately crop growth and yields. Motsi et al. [38] in Zimbabwe found that tied ridges retained significantly higher moisture than conventional tillage (ploughing) especially during the dry months. While Gicheru et al. [39], found that tied ridging conserved the lowest amount of moisture and attributed this to no runoff to impound and high evaporation losses due to increased soil surface area exposure in the semiarid areas of Laikipia district, Kenya. Gürsoy et al. [40] in semiarid Turkey found that ridge tillage accelerated drying of the seed zone; thus, low moisture contents were observed. Some water logging was observed in the LR 2012 season only and could explain the high moisture by HTR (15.61%) noted and hence the lowest moisture contents in the succeeding seasons (Table 2). Therefore, the effectiveness of tied ridges depends on the rainfall received and climatic conditions within a season.

The soil moisture decreased over time (WAP) during the four growing seasons (*P* < 0.05). Across the profile, the soil moisture was higher in the 20–40 cm than in the 0– 20 cm depth and varied among the different tillage practices in all seasons (*P* < 0.05). The changes in profile moisture content could be attributed to a combination of rainfall, soil evaporation, and transpiration or crop water uptake [25, 41, 42]. When the soil moisture content for each tillage method was averaged for the four seasons, a significant seasonal difference was found (*P* < 0.05) (Table 2). Soil moisture content by tillage was higher in OX > SSR > DPH > H > HTR > DP (*P* < 0.05). Fabrizzi et al. [43] reported an increase in soil moisture storage under conservation tillage due to decreases in evaporation, increases in the soil infiltration and the enhanced soil protection from rainfall impact. The average low soil moisture contents observed could be attributed to reduced water infiltration. This is due to the breaking of the large pores while the small pores are clogged by the dislocation of soil particles, which depend on tillage methods [44]. Soil moisture is the single most limiting factor to crop yields and thus tillage techniques that conserve moisture are important for increasing crop yields and limiting the devastating consequences of drought in semiarid areas [45].

There were no significant effects on soil moisture content due to the cropping system for all the seasons (*P* = 0.899) (Table 2). However, in SR 2012 and LR 2013, there were significant (*P* < 0.05) time × cropping systems interactions. From the results, there are inconsistencies observed by the cropping systems for each season on soil moisture contents that can be attributed to the varying crop requirements and climate conditions [46]. Soil water extraction by crops is determined not only by soil water content, evaporative demand and soil physical properties but also by physiological status of the crop [47] and could explain the nonsignificant effects observed.

### 3.2. Soil Surface Roughness

Soil surface roughness varied significantly (*P* < 0.05) over time in the growing seasons, between the tillage practices and across the different seasons (Table 3). A four-season summary shows a trend of HTR > SSR > DP > OX > DPH > H. Soil surface roughness soon after tillage was significantly higher among the tillage practices (*P* < 0.05) and decreased progressively in each season (Table 3). The HTR, SSR and DP had consistently higher values in all the four seasons with overall average values ranging from 27% to 66%.

The high surface roughness in HTR can be attributed to the raised ridges and basins created during tied ridging. The SSR plots had also higher surface roughness probably because of the furrows created during ripping. Ripping is usually done to open a narrow slit or furrow in the soil for sowing seeds while leaving unploughed sections between the planting rows [16]. The high values obtained in the DP plots could be attributed to the greater plough depth resulting in higher soil surface roughness which enhances greater infiltration primarily by surface retention of water in the small holes and depression. Soil surface roughness changes considerably with rainfall, wind, and cultivation events as indicated by Guzha [37], Mupangwa et al. [48], and Moreno et al. [49]. Lampurlanés and Cantero-Martínez [6] reviewed that surface roughness produced by tillage increases surface ponding, which allows rainwater to infiltrate into the soil, thus preventing runoff and increasing moisture retention in the soil profile. This explains why HTR had higher moisture levels in the LR 2012 season due to the water logging observed. Therefore, management practices aimed at adjusting the soil surface characteristics can promote soil processes that encourage soil moisture storage within the root zone [49, 50].

### 3.3. Crust Strength

Crust strength was affected significantly (*P* < 0.05) by the time of measurement, tillage and the growing season. There were also significant interactions of time × season, tillage × season, and time × tillage × season (*P* < 0.05) observed. The cropping systems did not influence the crust strength in the three seasons (*P* = 0.379).

Increase in crust strength as a season progressed was noted (Table 4) and could be attributed to natural formation of crust under raindrop impact since there was minimal human interference, only during weeding and data collection. Most soils in sub-Saharan Africa suffer from poor physical and chemical properties, which, combined with intensive rainfall events, make them particularly sensitive to crust formation [24, 37].

The average trend of crust strength observed during the SR 2012 season was in the order of SSR > OX > DPH > H > DP > HTR with values ranging from 1.03 to 1.18 MPa (Table 4). The crust formed on the ridges was weaker than other tillage practices as the season progressed. A probable cause of this was the inversion and mixing of top soil when constructing the tied ridges and this could have affected the structure of the top soil. However, at the base of the ridge basins, the crust strength was higher in all the seasons because of the excessive drying of the soil in the basin owing to direct insolation.

The crust strength trend observed during the LR 2013 season was in the decreasing order of SSR > OX > DP > HTR > H > DPH with average values ranging from 1.42 to 1.50 MPa (Table 4). Lower crust strength in H and DPH could be due to the loosening effect of the tillage used.

During the SR 2013 season, a trend of SSR > OX > H > DP > DPH > HTR was observed with values ranging from 1.14 to 1.22 MPa. A three-season average trend shows a decreasing trend of SSR > OX > H > DP > DPH > HTR with values ranging from 1.21 to 1.30 MPa. These results are in line with those reported by Khurshid et al. [51] where soil under conventional tillage (use of rigger in ridge tillage and use of a cultivator in deep tillage plots) had lower penetration resistance than minimum tillage (dibbling) treatments in the semiarid Faisalabad, Pakistan. Therefore, intensive tillage as observed in DP, DPH, and HTR causes a breakdown of soil surface sealing resulting in the weak crust strength observed (Table 4). The high crust strength observed in the SSR plots could be attributed to nonploughing of the soils between the planting rows [16]. This premise is supported by Miriti et al. [52] who observed higher crust in subsoiling-ripping (0.47 MPa) followed by tied ridging (0.40 MPa) and ox-ploughing (0.15 MPa), respectively, working in Makueni district, Eastern Kenya. The cropping systems did not significantly affect (*P* = 0.379) the crust strength but an overall average trend of intercrop (1.263 MPa) > sole maize (1.258 MPa) > sole bean (1.251 MPa) was noted.

Penetration resistance decreased with increases in soil moisture content and vice versa [53]. Penetrometer readings greater than 2 MPa are generally reported to produce a significant root growth reduction [54] which was not observed in this study. According to Lampurlanés and Cantero-Martínez [11], penetration resistance measured with a penetrometer is usually two to eight times greater than that actually undergone by the root tip, owing to the different way in which roots and probes penetrate the soil. However, roots can grow at a speed greater than the penetrometer reading because they can elongate through the biopores and interaggregate spaces [11]. Thus, the crust strength obtained in this study in all treatments and seasons is not expected to limit root penetration and plant growth.

### 3.4. Bulk Density, Porosity and Saturated Hydraulic Conductivity (*K*_sat_)

The bulk densities of the soils ranged from 1.20 Mg m^−3^ to 1.42 Mg m^−3^ across the seasons which are within the acceptable range for a clay loam (Table 1) [55]. The current study shows variation across the seasons (*P* < 0.05) (Table 5). Though not significant (*P* = 0.312), the average bulk density trend observed was DPH > SSR > H > OX > HTR > DP. The cropping systems did not influence the bulk densities in the four seasons (*P* = 0.502).

In the SR 2012 season, the bulk density increased (1.34 Mg m^−3^) at the end of the season from 1.27 Mg m^−3^ at the beginning of that season, while, in the SR 2013, higher bulk density (1.55 Mg m^−3^) was noted at the beginning of SR 2013 and was lower at the end of that season (1.35 Mg m^−3^). According to Husnjak et al. [56], tillage at the beginning of the growing season temporarily decreases soil bulk density but subsequent trips in the field for agronomic practices, rainfall events, and other disturbances activities can recompact the soil. Lower bulk density at the end of the growing season could be attributed to the short term loosening effect of the tillage method used.

The high densities in DPH and SSR could be attributed to the second passes of soil manipulation (harrowing and ripping) to the initial tillage method of disc ploughing and subsoiling in the respective plots. Agbede [57] found high bulk densities in the ploughing plus harrowing plots and attributed that to tractor wheel traffic and implement passes and lower macroporosity. The plough layer gets compacted as the tillage implement keeps passing the same depth season after season, thus increasing the bulk density. Eventually, such structural degradation can lead to a formation of a surface seal that further reduces infiltration and hinders seed germination [58]. The increase in bulk density in the SSR plots especially at the beginning of a season could also be attributed to the compacted unploughed sections during the land preparation.

Nonsignificance of tillage effect on bulk density over time has also been observed in other studies by Lampurlanés and Cantero-Martínez [11], Anken et al. [59], Osunbitan et al. [60], and Jabro et al. [61] due to different tillage practices. In contrast, Khan et al. [62, 63] reported higher bulk density with minimum tillage and lower bulk density with deep tillage. McVay et al. [45] reported that bulk density was greater in no-till than conventional tillage in a long term study at Manhattan and Ashland Bottoms sites in Kansas, USA. The bulk density is dependent on soil texture and the densities of soil minerals as well as their packing arrangement [58]. Generally, bulk density is not affected much by tillage practices [60]. Gomez et al. [64] realized that it took five years before changes in some of the soil physical properties (structure and aggregate stability, which are indicators of bulk density) could be detected as a result of the soil management practices. Full effect of tillage is observed after four to five years and this could not be obtained in our short term study.

Total porosity values of the soils varied across the seasons (*P* < 0.05) and ranged from 42 to 52% in all the seasons (Table 5). Though not significant, the tillage trend (*P* = 0.312) showed that conventional tillage plots had numerically greater porosity due to the lower bulk density achieved by the tillage method used. Soils with <40% total pore space are liable to restrict root growth due to excessive strength [58]. However, in this study, soil strength measured is not expected to restrict root growth. According to Gardner et al. [10], conventional tillage improved soil porosity by increasing the macroporosity. Since porosity is calculated from the relation between bulk density and particle density of soil, it is very much influenced by the soil bulk density as the particle density is not greatly altered by agricultural manipulations [65]. For any given soil, the higher the bulk densities, the more compacted the soil and the less the pore space as also observed in this study. This also affects the soil water transmission properties [66, 67].

Cropping system did not show a significant influence on the porosity (*P* = 0.502) but sole bean and maize showed higher porosity values than the intercrop (Table 5). This contrasts with results by Cheng and Coleman [68] and Fan et al. [69] who found an increase in porosity after intercropping and attributed that to increased root biomass and the stimulatory effects of the living roots on microbial activities that enhance soil organic matter decomposition. The distribution of roots in the profile thus determines the plants ability to absorb soil moisture.

The *K*_sat⁡_ values varied across the season (*P* < 0.05) and were very slow (<0.8 cm/h) in LR 2012, slow (0.8–2.0 cm/h) in SR 2012, and very slow (<0.8 cm/h) in LR 2013 and SR 2013 [55] (Table 5). Tillage did not significantly affect *K*_sat⁡_ values (*P* = 0.408) but an average trend of *K*_sat⁡_ among tillage treatments was HTR > OX > DPH > DP > H > SSR with values ranging from 0.66 cm/h to 0.98 cm/h. There were no significant interactions observed among the cropping systems (*P* = 0.744). The low *K*_sat⁡_ values in SSR plots have been reported elsewhere by Miriti et al. [52] in Makueni district, Kenya, who attributed it to the restricted lateral movement of water due to the low porosity in the unploughed sections.

Non-significant results on saturated hydraulic conductivity (*K*_sat⁡_) over time have also been observed by Chang and Lindwall [70] after 20 years of tillage in a clay loam soil in Alberta. Furthermore, inversion tillage (ploughing) makes the aggregates unstable during wetting which could cause lower *K*_sat⁡_. Pikul Jr and Aase [71] demonstrated that continuous tillage for 11 years in a dryland site in Eastern Montana, USA, resulted in low *K*_sat⁡_ due to a compacted 10–15 cm layer that impeded water movement. A soil profile description done at this study site showed a high bulk density of 1.54 Mg/m^3^ in the Ap horizon (0–10 cm depth) that impeded water infiltration as indicated by the low *K*_sat⁡_ value of 0.37 cm/h obtained.

Green et al. [72] also reported that the effects of tillage on the soil hydraulic properties under different tillage treatments are not always consistent across locations, soils and experiment designs. The overall low *K*_sat⁡_ values observed imply low infiltration rates and thus low rainwater intake by the soil [58]. The low *K*_sat⁡_ values could also be influenced by the soil particle size which is reflected in the texture of the soil [57]. This soil has a clay loam texture (39% clay), which has smaller grains and thus lower hydraulic conductivity [67]. These low values observed in this study also indicate the presence of a hardpan and thus deep subsoiling is required to improve the soil water uptake [55, 71].

## 4. Conclusion

Soil moisture conservation is important during land preparation and crop growth due to climate change, which affects the amount of rainfall and the rainfall seasons. The soil moisture trends by tillage and cropping systems were inconsistent in each cropping season. However, on average, the OX, SSR, and the DPH conserved the highest moisture. Nonsignificant results by tillage and cropping systems were also observed on bulk density, porosity and *K*_sat⁡_ values. The DP, HTR, and OX plots had numerically greater porosity (>45%) due to the lower bulk density achieved by the tillage method used. Based on the soil physical properties measured in this study, there was no significant advantage of a tillage practice over the other as the soil physical properties developed slowly after initiation of tillage practices. This suggests that long term tillage experiments (>4 seasons) under similar environmental and soil conditions are required to detect changes in soil physical properties as a result of the soil management practices. The long term studies will thus provide site-specific recommendations of the appropriate tillage practices for adoption in these semiarid areas.

## Figures and Tables

**Table 1:** Baseline chemical and physical properties of the experimental site (0–30 cm).

Soil property	Values
pH (H_2_O)	6.50
pH (0.01 M CaCl_2_)	5.61
%C	1.10
%N	0.09
K cmol/kg	2.35
Na cmol/kg	0.46
Ca cmol/kg	2.31
Mg cmol/kg	0.39
CEC cmol/kg	6.70
P (mg/kg)	13.50
Sand (%)	22.00
Silt (%)	39.00
Clay (%)	39.00
Textural class	Clay loam
Bulk density (g/cm^3^)	1.27
Saturated hydraulic conductivity (*K*_sat⁡_) (cm/h)	0.27
Saturation (cm/cm^3^)	0.664
Field capacity (cm/cm^3^)	0.508
Wilting point (cm/cm^3^)	0.480
Plant available water (cm/cm^3^)	0.028

**Table 2:** Soil moisture content (%) as affected by tillage, cropping systems, and depth (*n* = 4 seasons).

	Soil moisture (%)				
	LR 2012	SR 2012	LR 2013	SR 2013	Mean
Tillage					
Hand hoeing only (H)	14.57	13.74	9.63	15.39	13.33
Hand hoeing with tied ridges (HTR)	15.61	13.80	8.42	15.26	13.27
Disc ploughing (DP)	13.68	13.94	8.82	15.89	13.08
Disc ploughing + harrowing (DPH)	15.09	13.66	8.66	16.18	13.40
Ox-ploughing (OX)	14.55	15.33	9.98	16.55	14.10
Subsoiling-ripping (SSR)	—	14.35	9.96	15.98	13.43
Mean	14.70	14.14	9.25	15.87	13.44
Cropping system					
Sole bean	14.74	14.19	9.48	15.94	13.59
Sole maize	14.71	14.21	9.05	15.96	13.48
Intercrop	14.65	14.02	9.20	15.72	13.40
Depth					
0–20 cm	13.85	13.09	8.04	15.15	12.53
20–40 cm	15.55	15.18	10.44	16.59	14.44
Significance levels					
Time (weeks after planting)	<0.001	<0.001	<0.001	<0.001	<0.001
Tillage	0.019	0.158	0.003	<0.001	<0.001
Cropping systems	0.891	0.684	0.547	0.895	0.899
Depth	<0.001	<0.001	<0.001	0.003	<0.001

(—): not measured in that season.

**Table 3:** Effect of tillage on soil surface roughness (*n* = 4 seasons).

Tillage	Long rains (LR 2012)			Short rains (SR 2012)			Long rains (LR 2013)			Short rains (SR 2013)		
	At ploughing	Before planting	3 WAP	At ploughing	Before planting	3 WAP	At ploughing	Before planting	3 WAP	At ploughing	Before planting	3 WAP
H	36.8^ab^	31.8^b^	27.1^b^	30.7^a^	23.0^a^	16.2^a^	34.5^a^	25.5^a^	13.2^a^	39.0^a^	31.3^ab^	26.8^bc^
HTR	75.0^d^	72.5^d^	65.2^d^	67.8^d^	64.9^d^	58.6^c^	71.6^d^	62.6^d^	55.6^c^	72.4^c^	63.9^d^	56.0^d^
DP	53.7^c^	48.5^c^	40.7^c^	33.0^ab^	26.6^ab^	22.5^a^	36.8^ab^	27.8^ab^	19.5^a^	57.1^b^	49.8^c^	37.6^c^
DPH	29.8^a^	17.9^a^	13.5^a^	36.9^b^	33.7^b^	21.2^a^	40.7^b^	31.7^b^	18.2^a^	41.2^a^	28.7^a^	24.0^ab^
OX	48.1^bc^	38.7^b^	32.1^bc^	46.7^c^	32.0^b^	17.0^a^	50.5^c^	41.5^c^	14.0^a^	51.1^b^	42.1^bc^	14.7^a^
SSR	—	—	—	50.8^c^	47.2^c^	36.6^b^	54.6^c^	45.6^c^	33.6^b^	55.2^b^	45.1^c^	34.3^bc^
Mean	48.7	41.9	35.7	44.32	37.88	28.7	48.1	39.1	25.7	52.7	43.5	32.2
LSD (5%)	12.09	7.69	10.75	5.212	6.85	8.12	5.2	5.2	8.1	9.05	12.55	11.33
CV %	9.4	6.5	7.7	4.8	6.3	7.8	4.4	5.4	8.7	4.4	3.1	5.7

Tillage: H: hand hoeing only, HTR: hand hoeing with tied ridges, DP: disc ploughing, DPH: disc ploughing + harrowing, OX: ox-ploughing, SSR: subsoiling-ripping, and (—): not measured in that season.

Different letters within the columns indicate significant difference at 5% probability level.

**Table 4:** Soil crust strength as influenced by time of measurement, tillage practices, and the cropping systems (*n* = 3 seasons).

Soil crust strength (MPa)												
	Short rains 2012				Long rains 2013				Short rains 2013			
	3 WAP	7 WAP	10 WAP	15 WAP	4 WAP	6 WAP	10 WAP	16 WAP	4 WAP	8 WAP	12 WAP	17 WAP
Tillage												
H	1.034^a^	1.001	1.272	1.269	1.187^a^	1.553	1.574	1.469	1.187^a^	1.350	1.468	0.747^ab^
HTR	0.905^b^	1.007	1.088	1.124	1.133^a^	1.494	1.680	1.526	1.133^b^	1.282	1.462	0.672^c^
DP	1.111^a^	1.055	1.208	1.193	1.188^a^	1.475	1.708	1.472	1.188^a^	1.341	1.415	0.728^bc^
DPH	1.094^a^	1.023	1.255	1.217	1.136^a^	1.460	1.671	1.451	1.136^b^	1.301	1.417	0.769^ab^
OX	1.136^a^	1.025	1.256	1.234	1.175^a^	1.561	1.673	1.478	1.175^ab^	1.355	1.453	0.807^a^
SSR	1.111^a^	1.067	1.230	1.296	1.209^a^	1.568	1.705	1.519	1.209^a^	1.357	1.480	0.815^a^
LSD 5%	0.111	0.106	0.162	0.088	0.047	0.105	0.108	0.069	0.047	0.067	0.053	0.072
Cropping system												
Sole bean	1.026	1.032	1.221	1.241	1.177	1.529	1.617^b^	1.479	1.177	1.314	1.441	0.756
Sole maize	1.086	1.006	1.208	1.204	1.161	1.530	1.713^a^	1.482	1.161	1.338	1.462	0.749
Intercrop	1.085	1.051	1.225	1.222	1.176	1.496	1.675^a^	1.496	1.176	1.340	1.445	0.765
LSD 5%	0.072	0.064	0.052	0.036	0.051	0.046	0.047	0.036	0.051	0.041	0.029	0.048
Significance levels												
Tillage	0.005	0.741	0.218	0.012	0.018	0.165	0.160	0.193	0.018	0.129	0.085	0.007
Cropping system	0.171	0.369	0.794	0.131	0.773	0.245	<0.001	0.571	0.773	0.374	0.272	0.798
Tillage × cropping system	0.466	0.493	0.406	0.043	0.672	0.151	0.789	0.576	0.672	0.266	0.278	0.935
CV%	3.2	6.9	5.2	4.2	1.6	5.0	1.7	2.8	1.6	1.5	0.9	10.2

Tillage: H: hand hoeing only, HTR: hand hoeing with tied ridges, DP: disc ploughing, DPH: disc ploughing + harrowing, OX: ox-ploughing, and SSR: subsoiling-ripping.

Different letters within the columns indicate significant difference at 5% probability level.

**Table 5:** Effect of tillage practices and cropping systems on soil bulk density, porosity, and saturated hydraulic conductivity (*K*_sat⁡_).

	Bulk density (Mg/m^3^)	Porosity (%)	*K*_sat⁡_ (cm/h)
Time/season			
LR 2012^b^	1.43	46.19	0.65
SR 2012^a^	1.27	51.95	1.79
SR 2012^b^	1.34	49.32	1.01
LR 2013^a^	1.42	46.41	0.27
SR 2013^a^	1.55	41.39	0.36
SR 2013^b^	1.35	49.15	0.54
Tillage			
Hand hoeing only (H)	1.40	47.26	0.69
Hand hoeing with tied ridges (HTR)	1.39	47.75	0.98
Disc ploughing (DP)	1.37	48.46	0.71
Disc ploughing + harrowing (DPH)	1.41	46.71	0.77
Ox-ploughing (OX)	1.39	47.42	0.82
Subsoiling-ripping (SSR)	1.41	46.94	0.66
Mean	1.40	47.42	0.77
Cropping system			
Sole bean	1.38	47.77	0.83
Sole maize	0.39	47.45	0.74
Intercrop	1.40	47.09	0.76
Significance levels			
Time	<0.001	<0.001	<0.001
Tillage	0.312	0.312	0.408
Cropping system	0.502	0.502	0.744
CV%	9.15	10.14	25.52

^a^Beginning of season; ^b^at harvest.
